# Ondansetron: recommended antiemetics for patients with acute pancreatitis? a population-based study

**DOI:** 10.3389/fphar.2023.1155391

**Published:** 2023-05-10

**Authors:** Ge Wu, Yifei Ma, Wanzhen Wei, Jiahui Zeng, Yimin Han, Yiqun Song, Zheng Wang, Weikun Qian

**Affiliations:** ^1^ Department of General Practice, The First Affiliated Hospital of Xi’an Medical University, Xi’an, China; ^2^ Department of Hepatobiliary Surgery, The First Affiliated Hospital of Xi’an Jiaotong University, Xi’an, China; ^3^ Pancreatic Disease Center of Xi’an Jiaotong University, Xi’an, China

**Keywords:** ondansetron, acute pancreatitis, MIMIC-IV, multiple outcomes, recommended dose

## Abstract

**Objective:** Ondansetron administration is a common antemetic of acute pancreatitis therapy in the intensive care unit (ICU), but its actual association with patients’ outcomes has not been confirmed. The study is aimed to determine whether the multiple outcomes of ICU patients with acute pancreatitis could benefit from ondansetron.

**Methods:** 1,030 acute pancreatitis patients diagnosed in 2008–2019 were extracted from the Medical Information Mart for Intensive Care (MIMIC)-IV database as our study cohort. The primary outcome we considered is the 90-day prognosis, and secondary outcomes included in-hospital survival and overall prognosis.

**Results:** In MIMIC-IV, 663 acute pancreatitis patients received ondansetron administration (OND group) during their hospitalization, while 367 patients did not (non-OND group). Patients in the OND group presented better in-hospital, 90-day, and overall survival curves than the non-OND group (log-rank test: in-hospital: *p* < 0.001, 90-day: *p* = 0.002, overall: *p* = 0.009). After including covariates, ondansetron was associated with better survival in patients with multiple outcomes (in-hospital: HR = 0.50, 90-day: HR = 0.63, overall: HR = 0.66), and the optimal dose inflection points were 7.8 mg, 4.9 mg, and 4.6 mg, respectively. The survival benefit of ondansetron was unique and stable in the multivariate analyses after consideration of metoclopramide, diphenhydramine, and prochlorperazine, which may also be used as antiemetics.

**Conclusion:** In ICU acute pancreatitis patients, ondansetron administration was associated with better 90-day outcomes, while results were similar in terms of in-hospital and overall outcomes, and the recommended minimum total dose might be suggested to be 4–8 mg.

## Introduction

The onset of acute pancreatitis (AP) is insidious, and the symptoms are complex and changeable (Lankisch et al., 2015). Most mild patients are usually cured within a week, but 20% of patients will eventually develop into moderate or even severe acute pancreatitis, with a high fatality rate (Boxhoorn et al., 2020). The main symptom of AP is persistent, poorly localized epigastric pain, some radiating to the back, while most patients will also suffer from nausea and vomiting. Patients with acute pancreatitis tend to experience vomiting early, severely, and frequently. And the epigastric pain does not relieve after vomiting (Mergener and Baillie, 1998). Severe vomiting might even lead to fluid loss and eventual tissue hypoperfusion. The current therapeutic principle of acute pancreatitis is early goal-directed fluid resuscitation, analgesia, and nutritional support (Boxhoorn et al., 2020). At the same time, symptomatic treatment of patients with nausea and vomiting is an unavoidable problem for clinicians, which can not only reduce fluid loss in severe acute pancreatitis but also significantly advance the time for patients to start enteral nutrition (Tenner et al., 2013). Therefore, the use of antiemetics should be considered.

Ondansetron is a selective serotonin 5-hydroxytryptamine-3 receptor (5-HT_3_ R) antagonist, well-established in patients with nausea and vomiting associated with cancer chemotherapy, radiotherapy, anesthesia, and surgery (Wilde and Markham, 1996). It is also one of the most commonly used antiemetics in the ICU and emergency room (Athavale et al., 2020; Tao et al., 2021). Due to the close correlation between the 5-HT receptor and inflammation, many studies recently focused on the anti-inflammatory effect of ondansetron (Liu et al., 2011; Gong et al., 2019). One basic research has already confirmed that ondansetron could reduce pancreatic injury in the mice model of acute pancreatitis induced by cerulein (Tsukamoto et al., 2017). However, to date, no retrospective clinical study has analyzed the effects of ondansetron on multiple outcomes in ICU patients with acute pancreatitis to support this finding.

The good news is that the MIMIC-IV database, an extensive, open-access, long-term follow-up, and detailly-recorded patients information platform, allows us to analyze the multiple prognostic effects of ondansetron on ICU AP patients through real-world data and try to explore the optimal dose or medication time. In our preliminary study, we have already found that the proportion of ondansetron administration increased year by year from 2008 to 2019 for 4,060 initial admissions and 1,030 initial ICU admissions of patients with acute pancreatitis recorded in the MIMIC-IV database. As shown in Supplementary Figure S1, the proportion of ondansetron in ICU patients increased from 59.2% to 70.7% from 2008 to 2019. In general, it is essential to investigate the prognostic effects of ondansetron on ICU patients with acute pancreatitis and the possible more appropriate dose and timing.

## Methods

### Data source description

Our study cohort was extracted from the MIMIC database. The Medical Information Mart for Intensive Care (MIMIC) program is an extensive, single-center, and freely accessible clinical database hosted by the Laboratory for Computational Physiology at the Massachusetts Institute of Technology (MIT) (Johnson et al., 2022). The newly released MIMIC-IV (v2.0), updated on 12 June 2022, contains well-documented information on laboratory tests, medical behavior, and vital signs of 315,460 patients enrolled in Beth Israel Deaconess Medical Center (BIDMC), Boston, from 2008 to 2019 (Johnson et al., 2022). The most significant improvement over the previous version was the availability of out-of-hospital mortality from state death records, which allowed us to explore the impact of the intervention on the long-term outcome of patients, which was not covered by previous similar studies.

### Study population

**FIGURE 1:**
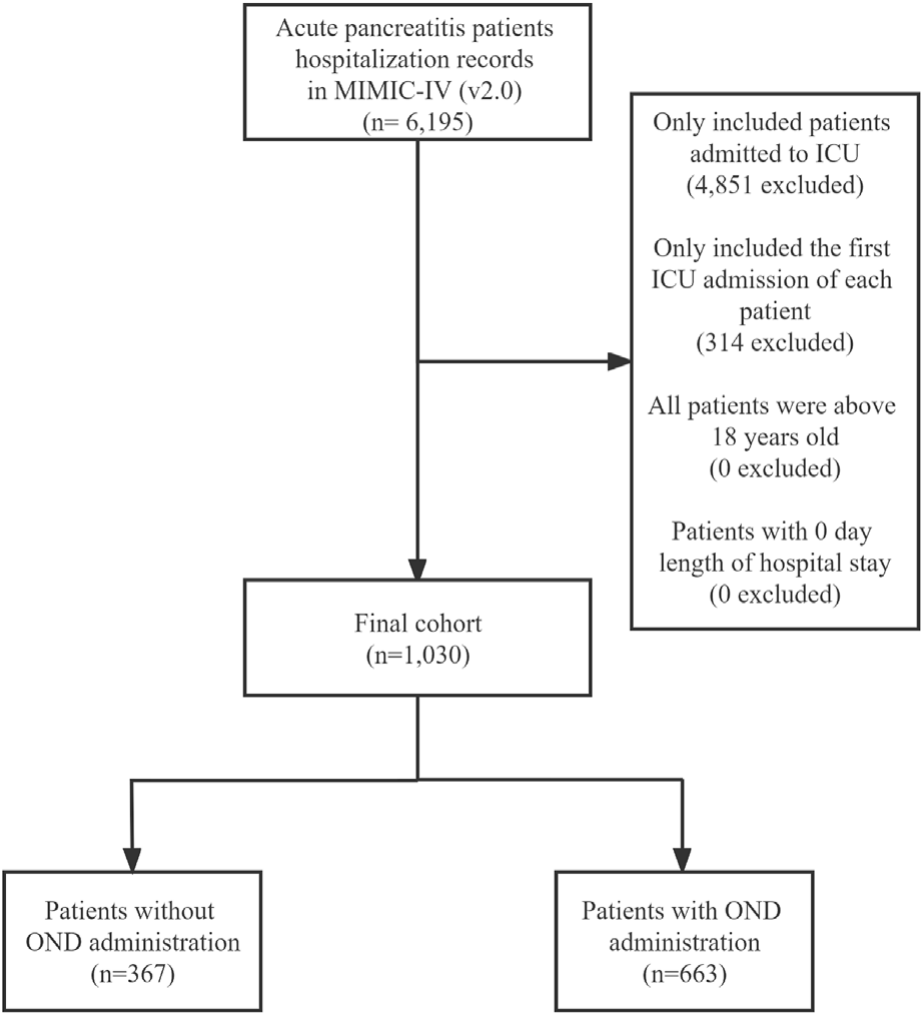
Study sample screening process of 1,030 acute pancreatitis patients from the MIMIC-IV database. ICU intensive care unit. OND ondansetron administration.

Patients whose diagnostic description included “acute pancreatitis” were enrolled in the study. A total of 6,195 hospitalization records of patients with acute pancreatitis were collected in the MIMIC-IV database. The patients who were not admitted to ICU were deleted, and only the first ICU records were kept. Finally, our study cohort determined 1,030 patients with acute pancreatitis during their first ICU admissions. The number of patients in each diagnosed title in the ICD (International Classification of Diseases) standard is shown in Supplementary Table S1. Patients were assigned to the “ondansetron administration group” (OND group) if all medication records for that hospitalization included at least one ondansetron administration record or to the “non-ondansetron administration group” (non-OND group) if they did not. The detailed screening process of the entire research cohort is shown in Figure 1.

### Variable extraction and outcomes

The demographic characteristics of patients we considered included the age of the admission, gender, admission period, and first measured weight. And the intervention records we focused on included renal replacement therapy (RRT) and mechanical ventilation (MV) during the first 24 h of ICU admission. Records of comorbidities enrolled congestive heart failure (CHF), chronic obstructive pulmonary disease (COPD), diabetes, and malignancy. In addition, the patient’s vital signs during the first 24 h in the ICU: mean heart rate, mean arterial pressure (MAP), mean respiratory rate, and mean body temperature were also considered. Laboratory tests were performed within the first 24 h, with values associated with the patient’s worst clinical status, including hemoglobin, platelets, white blood cells (WBC), hematocrit (HCT), alanine aminotransferase (ALT), creatinine, albumin, and lactate levels. Furthermore, we also determined the time point at which patients were first treated with ondansetron and the total dose of ondansetron during hospitalization in milligrams for subsequent sensitivity analysis.

Patient outcomes we studied included in-hospital survival, 90-day prognosis, and overall prognosis. Follow-up began on the day of admission and ended on the date of censored or MIMIC-IV (v2.0) updated.

### Statistical analysis

Continuous variables were described by medians with interquartile ranges (IQRs) and compared by *t*-test or Wilcoxon rank-sum test between groups. As for categorical variables, we used total numbers and percentages to present and compare the proportions by χ2 or Fisher exact tests. Moreover, standardized mean differences (SMD) were also used to represent the differences in variables between groups in both the original and matched cohorts. In terms of survival analysis, both in-hospital survival and overall prognosis could be extracted directly from the database, and the 90-day prognosis was calculated using total follow-up and overall status. After that, Kaplan-Meier (K-M) survival analyses were performed to generate curves of multiple outcomes and the log-rank tests to determine statistical differences between treatment groups. We further applied the multivariate Cox models to determine covariates’ independent effect on patient outcomes, including the administration of ondansetron. This model also served as the common basis for subsequent more complex multivariate analyses. Variables incorporated in multivariate analyses covered all mentioned above except the admission period. The numbers and percentages of missing data for each variable are shown in Supplementary Table S2. At the same time, we used multiple imputations to mitigate the estimation bias caused by missing data and assuming that data were missing randomly.

Imbalanced covariates between the intervention and control groups could lead to inaccurate multivariate analysis results, so we used the inverse probability of treatment weighting (IPTW) method to match the inter-group differences. This method calculated each patient’s weight to construct two virtual populations to balance constituent variables (Graffeo et al., 2019). In addition, we reconstructed a multivariate Cox model by treating ondansetron administration as a time-dependent covariate. The principle of this method is to treat the state of ondansetron patients before medication as the control group and to be included in the intervention group after medication to reduce the immortal bias of the traditional model effectively (Platt et al., 2019). Furthermore, we enrolled the restricted cubic spline (RCS) method to investigate the possible non-linear relationship between different drug doses or first medication timing and patient outcomes. Based on the multivariate Cox model, the method fits the non-linear relationship between continuous variables and patient outcomes by setting different knots, which could determine the vertex or inflection points according to the different shapes of the curve by the R package “rcssci,” thus providing more effective clinical guidance (Lv et al., 2018).

All our patients’ data from the database were extracted in SQL (Structured Query Language), and all statistical analyses were performed by Rstudio software (v4.2.2). Two-sided *p* < 0.05 was considered statistically significant.

## Results

### Patients baseline characteristics

In the ICU cohort we focused on for acute pancreatitis, a total of 663 patients received ondansetron during hospitalization (OND group), while the remaining 367 did not (non-OND group). Regarding demographic characteristics, patients in the OND group were younger (59 (46–72) vs. 62 (49–76); *p* = 0.014) and more female (55.5% vs. 62.1%; *p* = 0.046) than those in the non-OND group, and patients admitted between 2014 and 2019 appeared to be more likely to receive ondansetron (69.0% vs. 61.4%; *p* = 0.014). In terms of comorbidities and interventions, the OND group had a higher rate of malignancies (10.1% vs. 4.1%; *p* = 0.001) and lower rates of renal replacement therapy (4.8% vs. 9.3%; *p* = 0.008) and mechanical ventilation (28.2% vs. 45.5%; *p* < 0.001). As for laboratory tests, the OND group had a higher platelet level and lower creatinine. There was no statistical difference in other laboratory indicators. More detailed intergroup baselines are shown in Table 1.

**TABLE 1:** Baseline characteristics of the included patients from the MIMIC-IV database.

Covariates	MIMIC-IV (n = 1,030)				
	All patients	non-OND	OND	*p*-value	SMD
N	1,030	367	663		
Age	60 (47–73)	62 (49–76)	59 (46–72)	0.014	0.156
Male (%)	596/1,030 (57.9)	228/367 (62.1)	368/663 (55.5)	0.046	0.135
Weight (kg)	81.4 (69.4–98.7)	81.1 (68.5–98.8)	81.5 (70.0–98.7)	0.875	0.049
Admission period, n (%)				0.016	0.162
2008–2013	630/1,030 (61.2)	243/630 (38.6)	387/630 (61.4)		
2014–2019	400/1,030 (38.8)	124/400 (31.0)	276/400 (69.0)		
Interventions, n (%)					
RRT use (1st 24 h)	66/1,030 (6.4)	34/367 (9.3)	32/663 (4.8)	0.008	0.174
MV use (1st 24 h)	354/1,030 (34.4)	167/367 (45.5)	187/663 (28.2)	<0.001	0.365
Comorbidities, n (%)					
CHF	205/1,030 (19.9)	85/367 (23.2)	120/663 (18.1)	0.062	0.125
COPD	223/1,030 (21.7)	92/367 (25.1)	131/663 (19.8)	0.057	0.128
Diabetes	319/1,030 (31.0)	117/367 (31.9)	202/663 (30.5)	0.690	0.030
Malignancy	82/1,030 (8.0)	15/367 (4.1)	67/663 (10.1)	0.001	0.236
Vital signs					
Heart rate (bpm)	93 (80–107)	91 (78–104)	94 (81–108)	0.006	0.182
MAP (mmHg)	80.6 (72.4–91.1)	78.7 (70.8–87.7)	81.4 (73.4–91.7)	0.001	0.189
Respiratory rate (bpm)	20 (17–24)	20 (18–24)	20 (17–23)	0.254	0.059
Temperature (°C)	36.9 (36.7–37.3)	36.9 (36.6–37.3)	36.9 (36.7–37.3)	0.479	0.109
Laboratory tests					
Hemoglobin (g/dL)	10.4 (8.9–12.0)	10.5 (9.1–12.0)	10.3 (8.8–12.0)	0.682	0.003
Platelet (×10^9^/L)	167.0 (112.0–237.8)	152.0 (108.5–218.0)	174.0 (118.0–248.0)	0.003	0.134
WBC (×10^9^/L)	13.8 (9.8–19.6)	14.0 (9.8–20.4)	13.7 (9.8–19.0)	0.411	0.086
HCT (%)	31.5 (26.7–35.8)	31.7 (27.0–36.0)	31.4 (26.5–35.8)	0.707	0.007
ALT (IU/L)	57.0 (26.0–170.8)	61.0 (28.5–171.5)	55.0 (25.0–170.5)	0.235	0.051
Creatinine (mg/dL)	1.1 (0.8–2.1)	1.4 (0.9–2.5)	1.1 (0.7–1.8)	<0.001	0.245
Albumin (g/dL)	3.0 (2.5–3.5)	3.0 (2.5–3.5)	3.0 (2.5–3.5)	0.857	0.016
Lactate level (mmol/L)	1.9 (1.3–3.3)	1.9 (1.3–3.7)	1.9 (1.4–3.2)	0.504	0.070

*OND*, ondansetron administration; *SMD*, standardized mean differences; *RRT*, renal replacement therapy; *MV*, mechanical ventilation; *CHF*, congestive heart failure; *COPD*, chronic obstructive pulmonary disease; *MAP* mean arterial pressure; *WBC*, white blood cell; *HCT*, hematocrit; *ALT*, alanine aminotransferase.

### Survival differences

**FIGURE 2:**
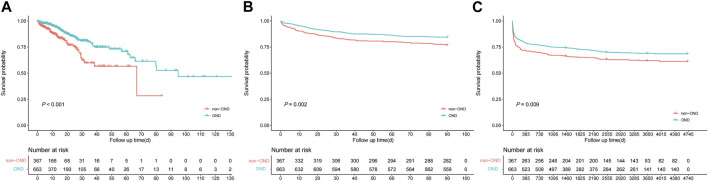
Kaplan-Meire survival curve analysis between treatment groups of multiple outcomes from the MIMIC-IV database. **(A)** in-hospital mortality **(B)** 90-day mortality **(C)** overall mortality.

Overall, the OND group had significantly better multiple outcomes than the non-OND group. First, the in-hospital mortality rate was 11.1% (74/663) in the OND group and 16.0% (59/367) in the non-OND group. Furthermore, the 90-day mortality rate was 15.6% (104/663) in the OND group and 23.1% (85/367) in the non-OND group. There was also a noticeable difference in overall survival between treatment groups at the end of follow-up (29.8% vs. 37.3%, *p* = 0.009). The K-M survival curves of treatment groups with different outcomes are shown in Figures 2A–C. Moreover, we performed IPTW matching between the two groups, and the inter-group baseline characteristics after matching are shown in Supplementary Table S3, which shows the algorithm balanced the inter-group differences of all variables well. The K-M curves after matching are shown in Supplementary Figure S2. We found a noticeable difference in in-hospital survival between groups. Although the results suggested that the 90-day and overall survival of the OND group were still better than that of the non-OND group, the difference was not statistically significant.

### Multivariate analysis

**FIGURE 3:**
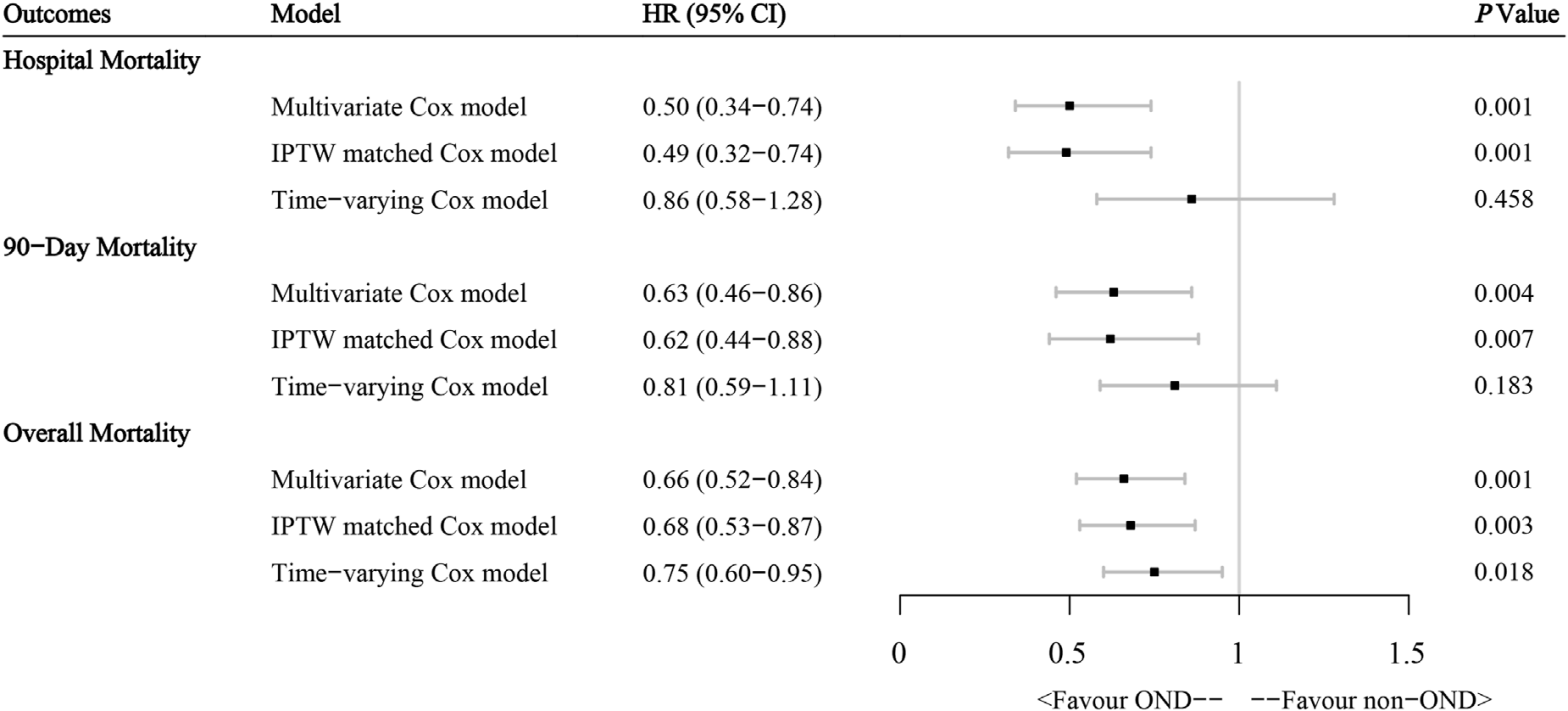
Effect of ondansetron administration on multiple outcomes in acute pancreatitis patients from the MIMIC-IV database through different multivariate Cox regression models. HR hazard ratio, IPTW inverse probability of treatment weighting.

We further tested the independent effect of ondansetron on outcomes in ICU AP patients in different multivariate models, as Figure 3 shows. First, in the original multivariate Cox model, the administration of ondansetron was considered an independent prognostic factor, with consistent benefits for in-hospital (HR: 0.50, 95% CI: 0.34–0.74, *p* = 0.001), 90-day (HR: 0.63, 95% CI: 0.46–0.86, *p* = 0.004), and overall (HR: 0.66, 95% CI: 0.52–0.84, *p* = 0.001) prognoses in ICU AP patients after the influence of other variables was balanced. These results were later confirmed in an IPTW-matched cohort with well-balanced baseline diversities, where ondansetron continued to benefit patients with multiple outcomes significantly. We added ondansetron into the multivariate Cox model as a time-dependent covariable to further verify the results. The results suggested that ondansetron still provided noticeable benefits to patients in terms of overall survival and tended to benefit in-hospital and 90-day survival, although not statistically significant.

In order to improve the robustness of the study results, we deleted all patients with missing data and finally obtained a cohort with complete data. The baseline characteristics of this patient cohort are shown in Supplementary Table S4. We repeated the above analysis for this cohort, and the results were similar, as shown in Supplementary Figure S3. In addition, the administration of metoclopramide, diphenhydramine, and prochlorperazine was also identified from all study patients’ medication records to exclude interference from the effects of other antiemetics. Our drug screening process was to include drugs with antiemetic primary pharmacological action in more than 50 prescriptions of all medications prescribed in the study cohort of 1030 ICU AP patients. Finally, the four drugs, including ondansetron, were eventually included in the study. After the collinearity problem of the model was eliminated, four commonly used antiemetics, including ondansetron, were enrolled in the multivariate Cox model, and the final results were shown in Supplementary Figure S4. After considering all drugs with an apparent clinical antiemetic effect, we found that ondansetron’s multiple prognostic benefits for ICU AP patients remained stable and were significantly superior to other antiemetics.

### Recommended application dose and time

**FIGURE 4:**
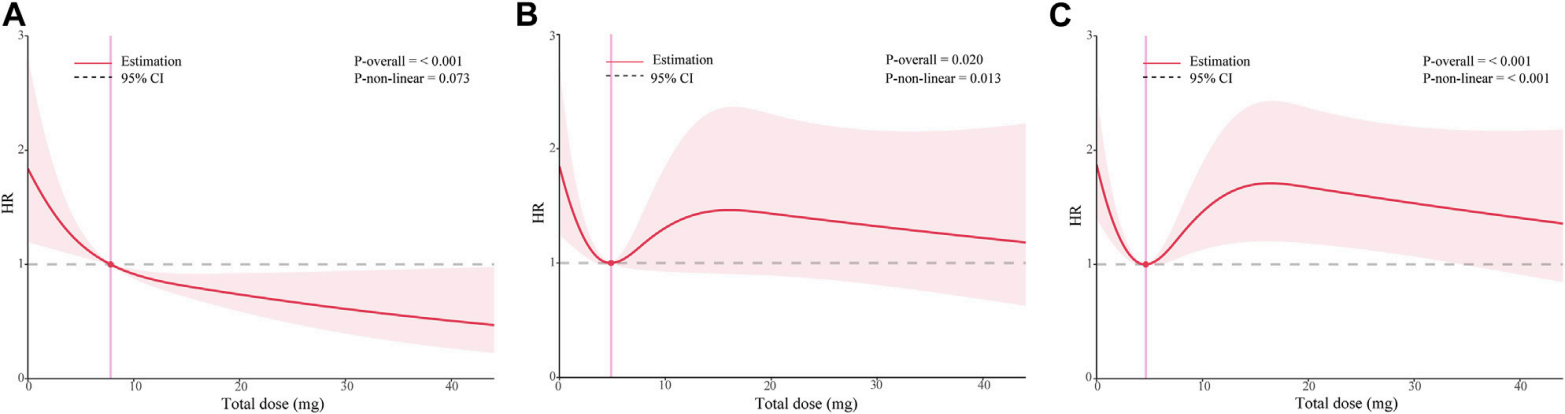
Dose-response curves of the relationship between ondansetron dose and multiple outcomes in patients with acute pancreatitis from the MIMIC-IV database. **(A)** in-hospital survival **(B)** 90-day prognosis **(C)** overall prognosis. HR hazard ratio.

To investigate the appropriate dose of ondansetron, we calculated the total dose for each patient during hospitalization in milligrams. We then replace the “1”used to refer to “intervention” in the traditional multivariate Cox model with the total ondansetron dose value, which is included in the restricted cubic spline analysis based on the multivariate Cox model. As Figure 4 shows, there are significant non-linear relationships between total doses of ondansetron and multiple outcomes in ICU patients with acute pancreatitis. The results suggested that the administration of 7.8 mg, 4.9 mg, and 4.6 mg ondansetron had the most significant survival benefit for in-hospital, 90-day, and overall outcomes, respectively, with the minimum dose. In addition, we also pay attention to whether the timing of administration is influential. As shown in Supplementary Figure S5, within the OND group, the time of initial ondansetron receipt and the interval between patient admission was not statistically associated with any of the three outcomes of interest.

## Discussion

In general, through a retrospective cohort of 1,030 acute pancreatitis patients diagnosed from 2008 to 2019 in the MIMIC-IV database, our study indicated for the first time that ondansetron administration could benefit in-hospital, 90-day, and overall outcomes of ICU AP patients. And the optimal doses were 7.8 mg, 4.9 mg, and 4.6 mg, respectively. Moreover, the survival benefit of ondansetron was not associated with the start time of medication. This result was robust in subsequent IPTW-matched cohort and sensitivity analyses. Therefore, we could conclude that ondansetron might be a recommended antiemetic for ICU patients with acute pancreatitis.

Ondansetron, a highly selective 5-HT_3_ receptor antagonist, was first used in the treatment of nausea and vomiting after chemoradiotherapy and anesthesia, which has also been widely used as an antiemetics in the emergency and ICUs at present (Wilde and Markham, 1996; Athavale et al., 2020). The mechanism of nausea and vomiting in acute pancreatitis is complex. At present, it is possible to infer that it starts from the stimulation of inflammation and toxins in the intestine, the secretion of the 5-HT_3_ receptor by the enteric chromaffin cells, and then passes into the vomiting center through the vagus nerve. Finally, the efferent fibers of the vomiting center are mainly through the vagus nerve to produce subsequent nausea and vomiting reflex (Heckroth et al., 2021). In addition to antiemesis, some studies found that antagonists of 5-HT_3_ R might also inhibit the incoming signals of the pancreatic vagus nerve and then reduce pancreatic secretion (Li et al., 2000; Mussa et al., 2008). Therefore, the application of ondansetron in ICU patients with acute pancreatitis has a plausible mechanism basis. As we suspected, acute pancreatitis patients in the MIMIC-IV database had a much higher proportion of ondansetron than the general ICU population (Fang et al., 2022), and the proportion showed an apparent upward trend from 2008 to 2019. However, retrospective clinical studies on the effect of ondansetron on the prognosis of ICU AP patients are still blank.

It is well known that the primary cause of death in patients with severe acute pancreatitis are systemic inflammation and severe organ failure (Boxhoorn et al., 2020). In recent years, many studies have focused on the anti-inflammatory effects of 5-HT_3_ receptor antagonists, which we consider one of the potential mechanisms by that ondansetron could provide survival benefits to ICU AP patients (Liu et al., 2011; Gong et al., 2019). One research found that ondansetron could reduce liver injury in rats with a hemorrhagic shock through the p38 mitogen-activated protein kinase (MAPK) dependent pathway (Liu et al., 2011). In addition, granisetron has been found to inhibit the accumulation of phosphorylated p38(P-p38) effectively and the transactivation of nuclear factor κB(NF-κB) in macrophages, protecting mice from death due to sepsis (Gong et al., 2019). More importantly, one basic research has found that ondansetron could reduce pancreatic injury in the cerulein-induced acute pancreatitis model (Tsukamoto et al., 2017). The authors randomly divided 33 mice with cerulein-induced acute pancreatitis into the control and experimental groups. They gave the experimental group a subcutaneous injection of 3 mg/Kg ondansetron. The blood levels of amylase, lipase, and interleukin (IL) −6 were determined, and histopathological grading of pancreatic injury was also performed. Finally, this study found that the blood indexes above were significantly reduced in the ondansetron injection group, and the inflammatory damage of pancreatic tissue was also alleviated. In addition to the anti-inflammatory effects described above, the study also speculated that ondansetron might reduce the secretion of pancreatic enzymes by acting on pancreatic acinar cells, thereby reducing blood amylase and lipase levels in mice. The mechanism may be related to the previously mentioned inhibition of pancreatic vagus signaling by 5-HT3 R antagonists, or it may result from decreased secretion of enzyme granules associated with 5-HT-dependent cytoskeletal dynamics (Sonda et al., 2013).

In addition, early stop emesis could effectively advance enteral nutrition’s start time (Tenner et al., 2013). A review of 11 RCTs showed that enteral nutrition initiated within 48 h of admission significantly reduced the risk of multiple organ failure, pancreatic complications, and death compared with parenteral nutrition (Petrov et al., 2009). Aside from the potential risk of dehydration associated with severe vomiting, the benefits of early enteral nutrition are also evident. In summary, we analyzed that ondansetron may provide survival benefits for patients with acute pancreatitis from various perspectives. These hypotheses support our main findings at various levels: ondansetron administration is associated with improved in-hospital, 90-day, and overall outcomes in ICU patients with acute pancreatitis.

In the research on the recommended dose of ondansetron, we found a clear inflection point at which the minimum dose can achieve sufficient clinical benefit for in-hospital, 90-day, and overall prognosis,7.8 mg, 4.9 mg, and 4.6 mg, respectively, similar to previous studies (Roila and Del, 1995; Hendren et al., 2015; Fang et al., 2022). In a retrospective study of all ICU patients in the MIMIC-IV database, moderate (8–16 mg) and low (0–8 mg) doses were associated with significant prognostic benefits for patients, but not high doses (Fang et al., 2022). Previous studies have shown that high doses of ondansetron have a significant potential risk of prolonged QTc secondary arrhythmia, which may be more significant in ICU patients receiving multiple medications (Kuryshev et al., 2000; Sutherland et al., 2022). Our study is the first to more clearly present the dose-response curve between ondansetron and three outcomes in ICU AP patients, suggesting that receiving a single dose (4 mg) of ondansetron may have achieved a near-maximum 90-day survival benefit in ICU AP patients. Of course, this conclusion needs to be confirmed by higher-quality research.

Furthermore, no statistical difference was found between the time point of administration and the outcome of patients in the OND group (Supplementary Figure S5), but combined with the benefits of early enteral nutrition, early control of vomiting symptoms may still be necessary. In a multivariate analysis that also considered several other commonly used antiemetics, we found that the prognostic benefits of ondansetron were prominent and stable compared with metoclopramide, diphenhydramine, and prochlorperazine, which was consistent with the conclusions of several previous studies (Crucitt et al., 1996; Koseoglu et al., 1998; Morris et al., 1998). Diphenhydramine is a first-generation antihistamine that antagonizes the H1 receptors (Hendren et al., 2015), and metoclopramide is a dopamine D2 receptor blocker that also acts centrally as peripherally (Camilleri and Shin, 2012). In addition, prochlorperazine is a phenothiazine, a dopamine receptor antagonist, and because of more side effects, which was no longer the first-line antiemetic drug (Gan et al., 2007). We speculate that the reason may be that the anti-inflammatory effect of Ondansetron as a 5-HT3 R antagonist is more significant in AP patients, not only in its antiemetic effect.

Our study still has limitations. First of all, as a retrospective study, even though we have enhanced the stability of the research results through various methods, there may still be variable interference that we cannot consider, so high-quality prospective research is still urgently needed. Secondly, due to the limitation of data sources, we cannot know patients’ real cause of death, so we can only use all-cause death as the outcome, which might be one-sided. In addition, because of the difficulty in obtaining the occurrence of side effects after administration, our dosage recommendations have limitations, and studies on ondansetron dosage need to be confirmed later. Nevertheless, this study is the most unambiguous indication yet of the effect of ondansetron on prognosis in patients with acute pancreatitis and can serve as an important basis for the principles of drug treatment in the ICU of acute pancreatitis.

## Conclusion

In ICU acute pancreatitis patients, ondansetron administration was associated with better 90-day outcomes, while results were similar in terms of in-hospital and overall outcomes, and the recommended minimum total dose might be suggested to be 4–8 mg. We recommend ondansetron as the drug of choice for ICU acute pancreatitis with nausea and vomiting.

## Data Availability

Publicly available datasets were analyzed in this study. This data can be found here: https://physionet.org/content/mimiciv/2.0/.
