# Supplementary material for: Ondansetron: recommended antiemetics for patients with acute pancreatitis? a population-based study
Source: Front Pharmacol. 2023 May 10;14:1155391. doi: 10.3389/fphar.2023.1155391 (PMC10205993; doi:10.3389/fphar.2023.1155391)
Supplement: Supplementary file 1 [file DataSheet1.docx]

**Ondansetron: Recommended antiemetics for Patients with acute pancreatitis? A population-based study**

Ge Wu^1^, Yifei Ma^2,3^, Wanzhen Wei^2,3^, Jiahui Zeng^2,3^, Yimin Han^2,3^, Yiqun Song^2,3^, Zheng Wang^2,3^ and Weikun Qian^2,3^

^1^ Department of General Practice, The First Affiliated Hospital of Xi'an Medical University, 48 West Fenghao Road, Xi'an, 710077, People's Republic of China

^2^ Department of Hepatobiliary Surgery, The First Affiliated Hospital of Xi'an Jiaotong University, 277 West Yanta Road, Xi'an, 710061, People's Republic of China

^3^ Pancreatic Disease Center of Xi'an Jiaotong University, 277 West Yanta Road, Xi'an, 710061, People's Republic of China

**Author Details**

First author: Ge Wu, Professor in the Department of General Practice, The First Affiliated Hospital of Xi'an Medical University. Email: [WG73212021@163.com](mailto:WG73212021@163.com), Address: 48 West Fenghao Road, Xi'an, 710077, Shaanxi Province, People's Republic of China,

Co-first author: Yifei Ma, Resident Doctor in Hepatobiliary Surgery, The First Affiliated Hospital of Xi'an Jiaotong University. Tel: (+86) 029-85324695, Email: mayiphy4628@stu.xjtu.edu.cn, ORCID: 0000-0001-5301-5562, Address: 277 Yanta Western Rd., Xi'an, 710061, Shaanxi Province, People's Republic of China,

Co-corresponding author: Zheng Wang, Prof, Ph.D., Professor in Hepatobiliary Surgery, The First Affiliated Hospital of Xi'an Jiaotong University, Tel: (+86) 029-85324695, Email: zheng.wang11@mail.xjtu.edu.cn, ORCID: 0000-0002-0490-466X, Address: 277 Yanta Western Rd., Xi'an, 710061, Shaanxi Province, People's Republic of China,

Corresponding author: Weikun Qian, Ph.D., Doctor in Hepatobiliary Surgery, The First Affiliated Hospital of Xi'an Jiaotong University. Tel: (+86) 029-85324695, Email: qianweikun@stu.xjtu.edu.cn, Address: 277 Yanta Western Rd., Xi'an, 710061, Shaanxi Province, People's Republic of China.

**Table S1: Number of patients in each diagnosed title of 1030 acute pancreatitis patients from the MIMIC-IV database in ICD standard**

| Diagnosis | ICD code | ICD version | No.of  patients |
| --- | --- | --- | --- |
| Acute pancreatitis | 5770 | 9 | 685 |
| Acute pancreatitis without necrosis or infection, unspecified | K8590 | 10 | 103 |
| Biliary acute pancreatitis without necrosis or infection | K8510 | 10 | 46 |
| Acute pancreatitis, unspecified | K859 | 10 | 36 |
| Alcohol induced acute pancreatitis without necrosis or infection | K8520 | 10 | 31 |
| Alcohol induced acute pancreatitis with uninfected necrosis | K8521 | 10 | 22 |
| Biliary acute pancreatitis | K851 | 10 | 21 |
| Alcohol induced acute pancreatitis | K852 | 10 | 16 |
| Acute pancreatitis with uninfected necrosis, unspecified | K8591 | 10 | 15 |
| Other acute pancreatitis without necrosis or infection | K8580 | 10 | 14 |
| Biliary acute pancreatitis with uninfected necrosis | K8511 | 10 | 9 |
| Biliary acute pancreatitis with infected necrosis | K8512 | 10 | 8 |
| Acute pancreatitis with infected necrosis, unspecified | K8592 | 10 | 7 |
| Idiopathic acute pancreatitis without necrosis or infection | K8500 | 10 | 4 |
| Drug induced acute pancreatitis without necrosis or infection | K8530 | 10 | 3 |
| Other acute pancreatitis | K858 | 10 | 3 |
| Alcohol induced acute pancreatitis with infected necrosis | K8522 | 10 | 2 |
| Drug induced acute pancreatitis | K853 | 10 | 2 |
| Drug induced acute pancreatitis with uninfected necrosis | K8531 | 10 | 1 |
| Idiopathic acute pancreatitis with infected necrosis | K8502 | 10 | 1 |
| Other acute pancreatitis with uninfected necrosis | K8581 | 10 | 1 |
| ICD: International Classification of Diseases | | | |

**Table S2: The information of missing data in the variables of interest of 1030 acute pancreatitis patients from the MIMIC-IV database**

| Variables | Missing, n (%) | |
| --- | --- | --- |
| Age | 0 (0) | |
| Gender | 0 (0) | |
| Weight (kg) | | 31 (3.0) |
| Admission period | | 0 (0) |
| Interventions |  | |
| RRT use (1^st^ 24h) | 0 (0) | |
| MV use (1^st^ 24h) | 0 (0) | |
| Comorbidities |  | |
| CHF | 0 (0) | |
| COPD | 0 (0) | |
| Diabetes | 0 (0) | |
| Malignancy | 0 (0) | |
| Vital signs |  | |
| Heart rate (bpm) | 2 (0.1) | |
| MAP (mmHg) | 2 (0.1) | |
| Respiratory rate (bpm) | 2 (0.1) | |
| Temperature (°C) | 14 (1.3) | |
| Laboratory tests |  | |
| Hemoglobin (g/dL) | 7 (0.6) | |
| Platelet (×10^9^/L) | 6 (0.5) | |
| WBC (×10^9^/L) | 7 (0.6) | |
| HCT (%) | 5 (0.4) | |
| ALT (IU/L) | 87 (8.4) | |
| Creatinine (mg/dL) | | 5 (0.4) |
| Albumin (g/dL) | 188 (18.2) | |
| Lactate level (mmol/L) | 244 (23.6) | |
| RRT renal replacement therapy, MV mechanical ventilation, CHF congestive heart failure, COPD chronic obstructive pulmonary disease, MAP mean arterial pressure, WBC white blood cell, HCT hematocrit, ALT alanine aminotransferase | | |

**Table S3: Baseline characteristics and SMD between treatment groups of acute pancreatitis patients from the MIMIC-IV database after IPTW matching**

| Covariates | **MIMIC-IV (n=1030)** | | | |
| --- | --- | --- | --- | --- |
|  | **non-OND** | **OND** | ***P* value** | **SMD** |
| N | 1033 | 1028 |  |  |
| Age | 59.2 (46.2-73.1) | 59.9 (47.3-72.7) | 0.773 | 0.022 |
| Male (%) | 600 (58.0) | 592 (57.6) | 0.902 | 0.009 |
| Weight (kg) | 79.0 (67.5-97.3) | 81.9 (70.0-99.0) | 0.228 | 0.018 |
| Admission period, n (%) |  |  | 0.835 | 0.015 |
| 2008-2013 | 638 (61.8) | 628 (61.1) |  |  |
| 2014-2019 | 395 (38.2) | 400 (38.9) |  |  |
| Interventions, n (%) |  |  |  |  |
| RRT use (1^st^ 24 h) | 66 ( 6.4) | 67 ( 6.5) | 0.951 | 0.004 |
| MV use (1^st^ 24 h) | 353 (34.2) | 351 (34.2) | 0.997 | <0.001 |
| Comorbidities, n (%) |  |  |  |  |
| CHF | 199 (19.3) | 203 (19.7) | 0.865 | 0.011 |
| COPD | 219 (21.2) | 222 (21.6) | 0.888 | 0.010 |
| Diabetes | 328 (31.7) | 317 (30.8) | 0.783 | 0.020 |
| Malignancy | 78 ( 7.5) | 82 ( 7.9) | 0.863 | 0.015 |
| Vital signs |  |  |  |  |
| Heart rate (bpm) | 93.8 (80.5-106.2) | 93.1 (79.7-107.4) | 0.805 | 0.016 |
| MAP (mmHg) | 80.9 (72.3-92.1) | 80.9 (72.8-91.1) | 0.919 | 0.018 |
| Respiratory rate (bpm) | 20.2 (17.6-23.5) | 20.2 (17.2-23.5) | 0.794 | 0.003 |
| Temperature (°C) | 37.0 (36.7-37.3) | 36.9 (36.6-37.3) | 0.379 | 0.007 |
| Laboratory tests |  |  |  |  |
| Hemoglobin (g/dL) | 10.5 (9.0-12.0) | 10.3 (8.8-12.0) | 0.729 | 0.003 |
| Platelet (×10^9^/L) | 157.2 (109.0-233.0) | 171.0 (114.0-240.0) | 0.252 | 0.005 |
| WBC (×10^9^/L) | 13.7 (9.6-20.2) | 13.9 (9.9-19.3) | 0.735 | 0.006 |
| HCT (%) | 31.7 (26.8-35.8) | 31.4 (26.5-35.7) | 0.767 | 0.003 |
| ALT (IU/L) | 56.0 (28.0-152.7) | 56.0 (26.0-179.0) | 0.993 | 0.006 |
| Creatinine (mg/dL) | 1.1 (0.8-2.1) | 1.1 (0.8-2.1) | 0.493 | 0.003 |
| Albumin (g/dL) | 3.0 (2.5-3.4) | 3.0 (2.5-3.5) | 0.925 | 0.016 |
| Lactate level (mmol/L) | 1.9 (1.3-3.3) | 2.0 (1.4-3.2) | 0.615 | 0.001 |
| OND ondansetron administration, SMD standardized mean differences, IPTW propensity score-based inverse probability of treatment weighting, RRT renal replacement therapy, MV mechanical ventilation, CHF congestive heart failure, COPD chronic obstructive pulmonary disease, MAP mean arterial pressure, WBC white blood cell, HCT hematocrit, ALT alanine aminotransferase | | | | |

**Table S4: Baseline characteristics and SMD between treatment groups of acute pancreatitis patients without missing data from the MIMIC-IV database**

| Covariates | **MIMIC-IV (n=618)** | | | |
| --- | --- | --- | --- | --- |
|  | **non-OND** | **OND** | ***P* value** | **SMD** |
| N | 233 | 385 |  |  |
| Age | 60.9 (48.9-73.2) | 58.2 (46.7-70.7) | 0.102 | 0.137 |
| Male (%) | 133 (57.1) | 214 (55.6) | 0.780 | 0.030 |
| Weight (kg) | 82.4 (68.1-98.2) | 82.5 (70.0-100.9) | 0.375 | 0.036 |
| Admission period, n (%) |  |  | 0.034 | 0.185 |
| 2008-2013 | 147 (63.1) | 208 (54.0) |  |  |
| 2014-2019 | 86 (36.9) | 177 (46.0) |  |  |
| Interventions, n (%) |  |  |  |  |
| RRT use (1^st^ 24 h) | 28 (12.0) | 27 ( 7.0) | 0.049 | 0.171 |
| MV use (1^st^ 24 h) | 130 (55.8) | 144 (37.4) | <0.001 | 0.375 |
| Comorbidities, n (%) |  |  |  |  |
| CHF | 50 (21.5) | 69 (17.9) | 0.329 | 0.089 |
| COPD | 53 (22.7) | 80 (20.8) | 0.634 | 0.048 |
| Diabetes | 71 (30.5) | 118 (30.6) | 1.000 | 0.004 |
| Malignancy | 8 ( 3.4) | 42 (10.9) | 0.002 | 0.293 |
| Vital signs |  |  |  |  |
| Heart rate (bpm) | 93.7 (82.5-106.1) | 99.7 (85.7-111.3) | 0.004 | 0.237 |
| MAP (mmHg) | 77.8 (70.6-86.5) | 81.3 (72.7-91.6) | 0.004 | 0.221 |
| Respiratory rate (bpm) | 20.9 (18.4-24.5) | 20.8 (17.6-24.4) | 0.468 | 0.050 |
| Temperature (°C) | 37.0 (36.7-37.4) | 37.0 (36.7-37.5) | 0.873 | 0.096 |
| Laboratory tests |  |  |  |  |
| Hemoglobin (g/dL) | 10.5 (9.1-11.9) | 10.3 (8.9-12.0) | 0.968 | 0.017 |
| Platelet (×10^9^/L) | 147.0 (101.0-217.0) | 167.0 (110.0-230.0) | 0.024 | 0.103 |
| WBC (×10^9^/L) | 14.7 (10.3-22.0) | 14.7 (10.3-19.6) | 0.441 | 0.112 |
| HCT (%) | 31.4 (27.0-35.9) | 31.2 (26.7-35.5) | 0.784 | 0.008 |
| ALT (IU/L) | 60.0 (27.0-163.0) | 67.0 (30.0-190.0) | 0.525 | 0.070 |
| Creatinine (mg/dL) | 1.6 (1.0-3.1) | 1.2 (0.8-2.3) | <0.001 | 0.210 |
| Albumin (g/dL) | 2.9 (2.4-3.4) | 2.9 (2.4-3.4) | 0.995 | 0.013 |
| Lactate level (mmol/L) | 2.2 (1.4-3.8) | 2.0 (1.4-3.4) | 0.185 | 0.079 |
| OND ondansetron administration, SMD standardized mean differences, IPTW propensity score-based inverse probability of treatment weighting, RRT renal replacement therapy, MV mechanical ventilation, CHF congestive heart failure, COPD chronic obstructive pulmonary disease, MAP mean arterial pressure, WBC white blood cell, HCT hematocrit, ALT alanine aminotransferase | | | | |

**
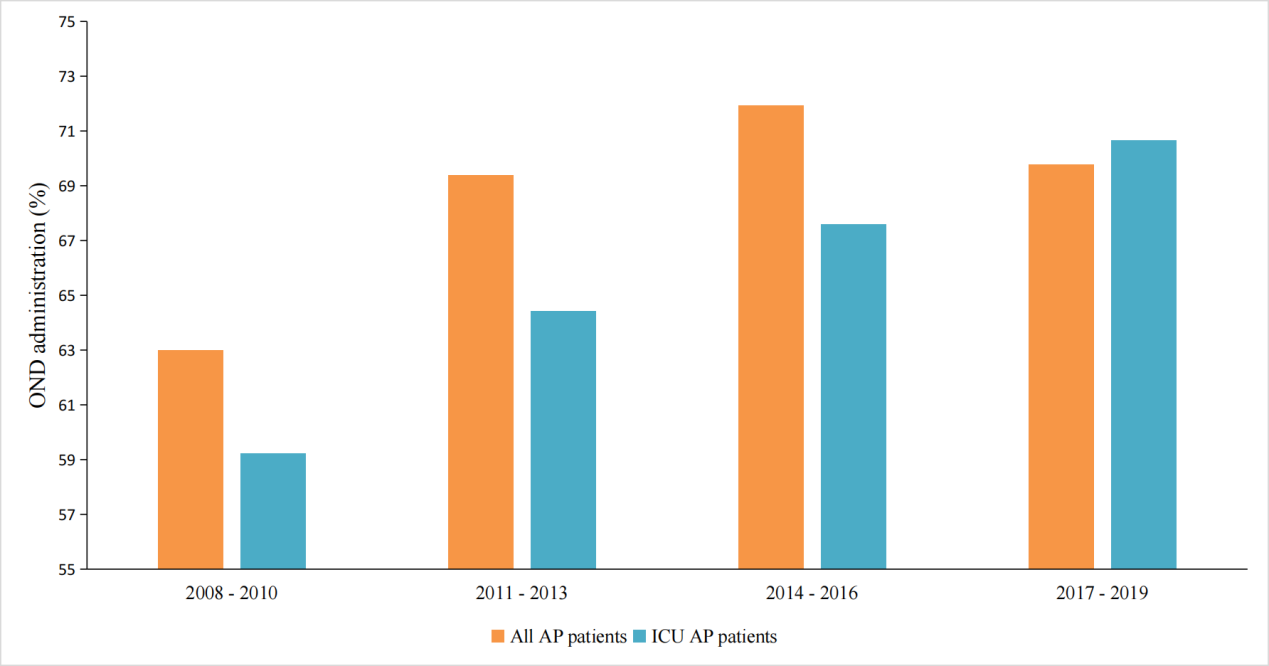
**

**Figure S1:** Trends in the proportion of acute pancreatitis patients treated with ondansetron who were first admitted to the ICU or the hospital from 2008 to 2019 in the MIMIC-IV database OND: ondansetron administration, AP: acute pancreatitis, ICU: intensive care unit

**
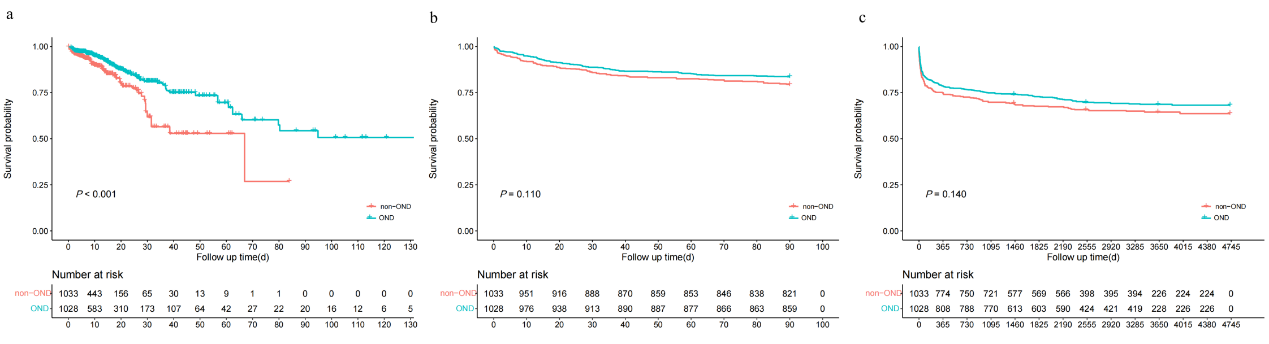
**

**Figure S2:** Kaplan-Meire survival curve analysis between treatment groups of multiple outcomes after IPTW matching from the MIMIC-IV database. **a** in-hospital mortality **b** 90-day mortality **c** overall mortality. OND: ondansetron administration, IPTW: propensity score-based inverse probability of treatment weighting.


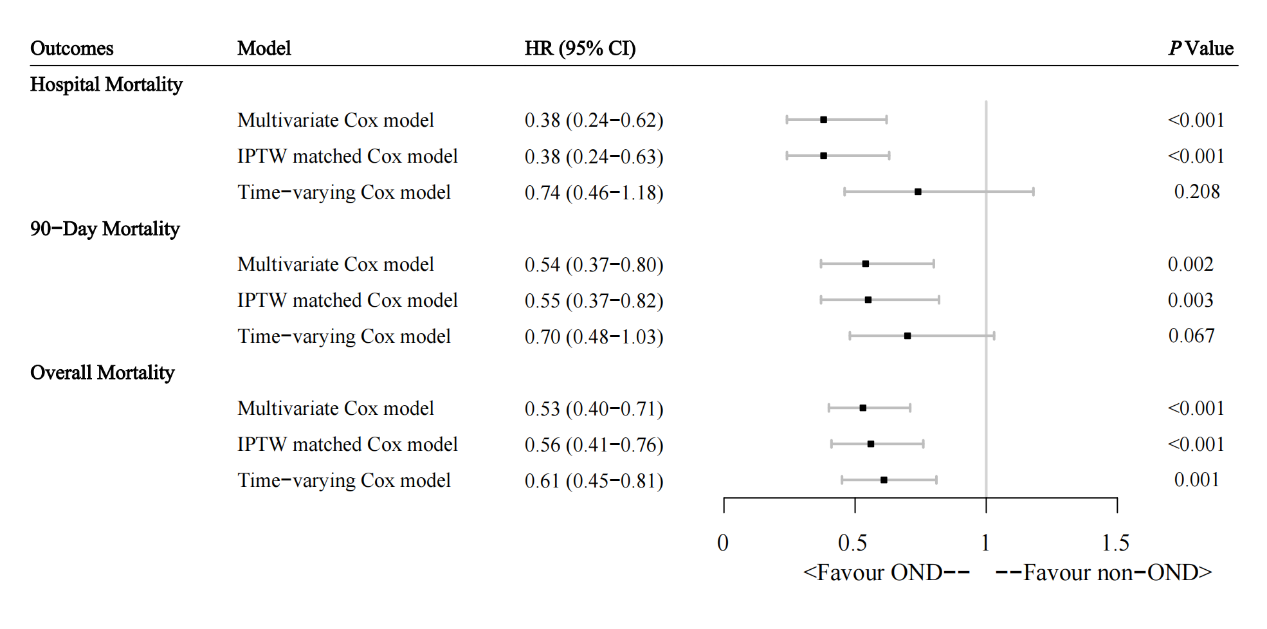


**Figure S3:** Effect of ondansetron administration on multiple outcomes in acute pancreatitis patients without missing data from the MIMIC-IV database through different multivariate Cox regression models. HR: hazard ratio, IPTW: inverse probability of treatment weighting


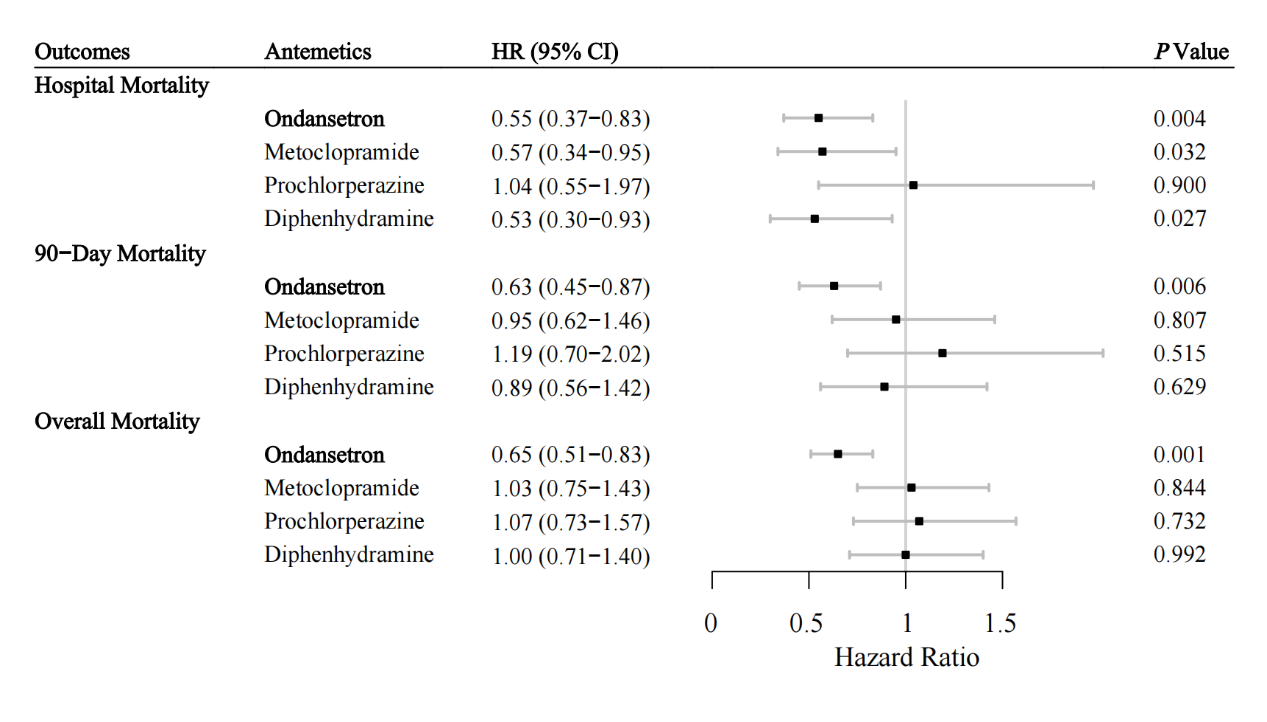


**Figure S4:** Multivariate cox regression analysis of prognostic effects of all commonly used antiemetic drugs in the MIMIC-IV database on ICU acute pancreatitis patients


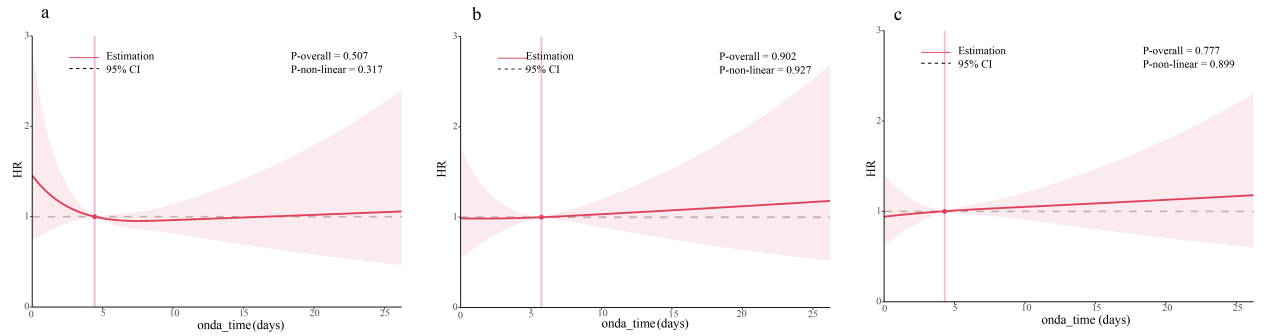


**Figure S5**: Dose-response curves of the relationship between the first medication time of ondansetron and multiple outcomes in patients with acute pancreatitis from the MIMIC-IV database. **a** in-hospital survival **b** 90-day prognosis **c** overall prognosis. HR hazard ratio
